# Transnasal delivery of human A-beta peptides elicits impaired learning and memory performance in wild type mice

**DOI:** 10.1186/s12868-016-0280-9

**Published:** 2016-07-04

**Authors:** Kristina Endres, Sven Reinhardt, Anastasia Geladaris, Julia Knies, Marcus Grimm, Tobias Hartmann, Ulrich Schmitt

**Affiliations:** Department of Psychiatry and Psychotherapy, University Medical Center, Johannes Gutenberg University, Untere Zahlbacher Straße 8, 55131 Mainz, Germany; Deutsches Institut für DemenzPrävention (DIDP), Neurodegeneration and Neurobiology, Saarland University, Homburg/Saar, Germany; Experimental Neurology, Saarland University, Homburg/Saar, Germany

**Keywords:** Amyloid precursor protein, Amyloid beta peptide, Alzheimer’s disease, Hippocampus, Amygdala, Memory task, Fear conditioning, Transnasal delivery

## Abstract

**Background:**

Murine models of Alzheimer’s disease (AD) are mainly based on overexpression of pathologic amyloid precursor protein and/or presenilins. Those genes resemble underlying cause of early onset type of AD while about 99 % of all human cases are to be characterized as sporadic, late onset. Appropriate animal models for this type of AD are still missing. We here investigated, if transnasal delivery of A-beta 42 peptides might serve to mimic pathological effects in mice.

**Results:**

A-beta 42 peptides, used for the behavioral study, showed the expected dose-dependent toxicity in neur oblastoma cell line SH-SY5Y and were able to form higher molecular weight species in vitro. Upon delivery into nostrils of wild type mice, protein bands that might represent aggregation products of the exogenously applied human A-beta 42 were only observed in total brain homogenates from mice pre-treated with mannitol. By using TAMRA-labeled A-beta 42 peptides we demonstrated, that transport throughout the brain was achieved already 1 h after administration. FVB/N mice treated with A-beta 42 for 3 days were significantly impaired in the cue-retention condition of the fear conditioning task as compared to controls whereas A-beta-treated C57B6/J mice were impaired in the context condition. In the Morris water maze test, these mice also displayed a delayed learning performance, indicated by significantly longer time to find the platform. Those deficits were also seen for memory performance in the probe trial as measured by number of crossings of the former platform position and time spent in the goal quadrant.

**Conclusions:**

Existing AD mouse models are of genetic origin and need prolonged housing time before onset of pathology. Our short-term treatment induced learning and memory deficits via exogenous application of A-beta peptides comparable to those observed for the transgenic animals. With the transnasal A-beta 42 treatment we present an approach to investigate purely A-beta related changes suitable as a model for symptoms of Alzheimer’s dementia (AD). Resulting behavioral deficits were indicative for familial type of Alzheimer’s disease as well as for the late onset variant.

**Electronic supplementary material:**

The online version of this article (doi:10.1186/s12868-016-0280-9) contains supplementary material, which is available to authorized users.

## Background

Brain aging is probably a multifaceted process in which small deviations from homeostasis might already have deleterious outcome. Alzheimer’s disease (AD) is one of the major disorders of the human brain occurring in the elderly and aging is the most prominent risk factor assigned to the sporadic form (or late onset form, LOAD) of this type of dementia. To mimic such a complex progressive disorder in animal models is rather difficult (e.g. [1]). Within the last decades, several mouse and also rat models have been established for familial AD (FAD), mainly based on the three genes that are involved in development of the subtype of the disease: single transgenic mice with either mutations in APP (amyloid precursor protein) or one of the presenilins (PS1 or 2) reveal some of the disease criteria such as plaque deposition or memory deficits. Double, triple or even quintuple transgenic mice have been established with combination of each gene to overcome low intensity and long time frame of progression of pathological hallmarks (for a characterization of 10 such models see: [2]). Adding mutated tau species in part completed the footprint of AD in the animal model [3]. Recently, a knock-in mouse with subtle overexpression of human BACE-1 has been described that seems to reflect certain characteristics of AD in a more physiological way than the multi-transgenic mice: while no monomeric A-beta was detectable, the toxic A-beta hexamer formed from endogenous APP cleavage products was observed and mice displayed cognitive impairment that was intensified by aging [4]. Other researchers suggest e.g. crossbreeding of traditional AD mouse models with insulin desensitized mouse strains to end up with a more complete picture of the disease [5]. All these models face the drawback of the need for prolonged housing times for the occurrence of appropriate pathological signs, production of non-transgenic littermates in case of heterozygous breeding and their origin by genetic manipulation. The latter might interfere with unforeseen metabolic or signaling pathways contributing to the resulting phenotype and a limited face validity with respect to late onset non familiar AD.

It is intensively discussed in the field, if A-beta peptide overproduction or reduced degradation is the main impetus of sporadic AD development (e.g. [6]). However, acute intracerebroventricular (icv) injections of synthetic A-beta 42 oligomers impaired consolidation of the long-term recognition memory [7]. Moreover, icv injection of the less toxic A-beta 40 species similarly impaired cognitive function as measured by Morris water maze testing [8]. As stereotactic injection is an invasive manipulation that also needs expertise of the investigators, a more mild and easy to handle technique of A-beta delivery into the brain would be helpful. Transnasal delivery of peptides has been repeatedly described in literature to be of success in mice (e.g. [9, 10]). This might be supported by the murine nasal cavity architecture featuring high surface area (e.g. [11]). To build up on that, the present investigation aimed at analyzing whether intranasal A-beta application could serve as a less invasive, less stressful lesion model for late onset related beta-amyloid pathology.

## Methods

### Material

Human A-beta 42 (W. M. Keck Foundation Biotechnology Resource Laboratory, Yale, UK) was prepared and purified as follows: 1 mg of lyophilized material was dissolved in 10 mM NH_4_OH and passed through a 0.22 µm filter (Ultrafree Durapore PVDF, Merck Millipore, Schwalbach, Germany) followed by 30 k filtration using Amicon devices (Merck Millipore, Schwalbach, Germany). Protein concentration was calculated via absorbance at 280 nm (Nanodrop, VWR International GmbH, Erlangen, Germany). Aliquots of the peptide were diluted 1:2 with PBS and stored at −80 °C. N-ethylmaleimide (NEM) was purchased from Calbiochem (Merck, Darmstadt, Germany) and mannitol from Sigma Aldrich (St. Louis, MO, USA).

### Cell culture and toxicity assays

The human neuroblastoma cell line SH-SY5Y was maintained at humidified air (95 %), 5 % CO_2_, 37 °C, and cultured in DMEM/F12 (Life Technologies, Darmstadt, Germany) supplemented with 10 % FCS and 1 % Glutamine (both GE Healthcare, Piscataway, NJ, USA). For assay conditions, cells were seeded at a density of 30,000 cells per well of a white glas-bottom 96 well plate (Greiner Bio-One GmbH, Frickenhausen, Germany) in OptiMEM (Life Technologies, Darmstadt, Germany). After 24 h incubation period, culture supernatant was aspirated and exchanged by 50 µl fresh OptiMEM or OptiMEM supplemented with A-beta peptides in NH_4_OH/PBS at the indicated concentration or with solvent. Cells were incubated for 24 h; subsequently, 5 µl cell supernatant for LDH release assay were pipetted to a clear 96 well plate and assay was performed as recommended by the manufacturer (Biovision, Milpitas, CA, USA). 5 µl MTT (5 mg/ml, Sigma Aldrich, St. Louis, MO, USA) were added per well to the cells. Development of formazane crystals was controlled by light microscopy and crystals lysed after 1 h of incubation [humidified air (95 %), 5 % CO_2_ and 37 °C] with 200 µl 0.1 N HCl in isopropanol per well by vigorous pipetting. Then, absorption was measured at 595 and 620 nm in a plate reader (Asys Hitech Expert 96, Biochrom, Cambridge, UK) and viability calculated by A_595_–A_620_ in % of control (solvent-treated cells).

### In vitro fibrillization of A-beta peptides

A-beta 42 peptides (132 µM) were fibrillized in a total volume of 25 µl 1xPBS for 24 h at 37 °C, 300 rpm in a thermomixer (Eppendorf AG, Hamburg, Germany). For the monomeric form, the buffer was incubated as described and A-beta was added immediately before preparation for western blotting.

### Animals

Male C57Bl6/J, FVB/N or abcb1a/1b^−/−^ mice (FVB/N background, Taconic, USA, [12]) and 5xFAD (Jackson Laboratory; [13]) stably cross-bred with C57Bl6/J mice from the animal facility of the University Medical Center of Mainz were used (body weight 25–45 g). Animals were housed in groups of 2–5 with free access to food and water. A 12 h light–dark cycle (6 a.m.–6 p.m. light on) was maintained at a temperature of 22 °C and a relative humidity of 60 %.

### Study design and drug administration

#### Intranasal administration

##### Acute treatment

Mice received A-beta 42 peptides in both nostrils after mild *i.p.* chloralhydrate anesthesia (0.006 ml/g bodyweight of a 7 % saline solution). Per administration maximally 5 µl solution at once were given with an intermission of 2 min to ensure proper breathing and absorption of the A-beta-solution. Thus, mice received 10 µg A-beta 42 in total. To facilitate penetration into the CNS, mice were pre-treated with mannitol. Mannitol was injected *i.p.* at a dose of 0.03 ml/g bodyweight [14] 20 min prior to the A-beta application. For prevention of A-beta degradation NEM (5 µl of 1 M NEM) was pipetted into each nostril 5 min prior to the first A-beta administration if indicated.

For fluorescence-signal based A-beta detection TAMRA-A-beta 42 (Anaspec, Seraing, Belgium) was administered as described above (5 µg per nostril). As a control, water in the appropriate volume supplemented with the dilution reagent from the vendor was used. 1 h after administration, animals were sacrificed and brains dissected in six slices (as given in Fig. 2b). Tissue was homogenized in 500 µl PBS per slice (Tissue lyzer, Qiagen, Hilden, Germany; 5 min 50 Hz), centrifuged at 4 °C 500 g for 10 min and supernatant diluted 1:5 in PBS. 80 µl homogenate were supplemented to fluorescence measurement (Exc 540/Em 580) in technical duplicates. Mean values obtained for respective tissue slice of the control treated animal were subtracted from the values from the A-beta treated mouse.

For immunohistochemistry, solvent or A-beta 42 (Anaspec, Seraing, Belgium) were administered as described above (5 µg per nostril) and animals sacrificed 1 h later.

##### Treatment for behavioral investigations

For analysis of behavioral effects, A-beta 42 peptides were administered for three consecutive days (day 1–3 of the MWM, day −1, 1, and 2 of the fear conditioning). Mice were pre-treated with mannitol as described for the acute treatment. Subsequently, A-beta/NH_4_OH/PBS-solution (10 µg peptide/day) or NH_4_OH/PBS alone (controls) were pipetted into nostrils as described before [minimal number of animals (n) = 5 per group, indicated in the respective figure].

#### Preparation of brain homogenates

Animals from acute treatment experiments were sacrificed 1 h after A-beta application under isoflurane anesthesia via decapitation. Brains were quickly removed and immediately stored on ice. If needed, tissue was stored at −80 °C until further use. Per hemisphere 400 µl ice cold homogenization buffer [20 mM Tris/HCl pH 8.5, supplemented with protease inhibitor mix (Complete Mini, Roche, Mannheim, Germany)] were added and tissue homogenized for 10 min at 50 Hz in a pre-chilled swing mill (Tissue lyzer, Qiagen, Hilden Germany) using 5 mm pre-chilled stainless steel beads (Qiagen, Hilden Germany). Homogenates were supplemented with formic acid (70 % final) and incubated at 4 °C for 1 h in a test tube rotator (GFL 3025, Burgwedel, Germany). Supernatant from ultracentrifugation (1 h 4 °C 35.000 rpm, rotor 70.1 Ti, Beckman Coulter, Krefeld, Germany) was neutralized with 20-fold volume of 1 M Tris. Protein content of samples was assessed by the method of Bradford (RotiNanoQuant, Carl Roth, Karlsruhe, Germany).

#### Western blotting

For tissue analysis, samples were adjusted with LDS NuPAGE buffer (1×, Life Technologies, Darmstadt, Germany) and DTT (1 M, 10 % v/v) to obtain 20 µg protein/15 µl and incubated for 10 min at 70 °C. Samples were subjected to 4–12 % NuPAGE gradient gel (Life Technologies, Darmstadt, Germany) using MES buffer and a voltage of max. 150 V. For in vitro fibrillization analysis, peptide preparations were supplemented with LDS NuPAGE buffer (1×) and DTT (1 M, 10 % v/v) and boiled for 5 min at 95 °C. Samples were separated on 10 % SDS–polyacrylamide gels and blotted onto nitrocellulose membrane at 100 V for 2 h. Immunodetection of APP and A-beta was carried out by blocking the membranes for 1 h in blocking solution [0.2 % I-Block (Life Technologies, Darmstadt, Germany), 0.05 % Tween 20 (AppliChem, Darmstadt, Germany) in PBS] and incubation overnight at 4 °C with antibody 6E10 (Covance, Munich, Germany) at a dilution of 1:1000 in blocking solution. Blots were incubated with anti-mouse secondary antibody coupled with horseradish peroxidase (Thermo Scientific, Karlsruhe, Germany) and signals obtained by applying SuperSignal West Femto chemiluminescent substrate (Thermo Scientific, Karlsruhe, Germany) were detected using a CCD-camera imaging system (Stella Camera, Raytest, Straubenhardt, Germany).

### ELISA

Human A-beta 42 ELISA was performed as recommended by the manufacturer (IBL, Hamburg, Germany). Samples from a 5xFAD mouse brain were used as a positive control.

#### Immunohistochemistry

Brains were dissected, washed with 0.9 % NaCl and immediately submerged in 4.5 % formalin for 24 h at RT. Subsequently, hemispheres were embedded in paraffin at 58 °C and cut in 2 µm thick tissue sections using a rotary microtome (Leica RM 2245). After mounting on histological slides (Superfrost plus, Menzel), sections were stained following standard protocols using the primary antibody 6E10 (diluted 1:500 in Antigen Retrieval Buffer 1; Medac, Wedel, Germany) and DAB as the chromogenic substrate. Microscopic pictures were taken with 4 × 10 or 10 × 10 magnification (EVOS XL, Life Technologies, Darmstadt, Germany).

### Morris water maze

Spatial learning and memory was tested by the Morris water maze hidden platform task in C57Bl6/J mice using the maze and protocol as described previously [15]. In brief, the platform stayed in the same quadrant for all trials (South West) and the animals were released from four different positions at the pool perimeter. Mice performed four trials per day on five consecutive days with a maximum length of 60 s per trial and an inter-trial interval of 90 s. Mice were allowed to stay on the platform for 10 s. On the sixth day, a probe trial (60 s) without platform was performed (starting position: North East). Learning was assessed by measuring the latency to find the platform. For characterization of memory the number of potential platform crossings and % of goal quadrant occupancy during the probe trial were assessed.

### Fear conditioning

Fear memory was tested in an auditory fear conditioning task (see Fig. 3a) similarly to: [16]. Mice underwent three sessions: (1) A single conditioning session with one tone presentation (30 s, 9 kHz, 75 dB SPL, pulsed 5 Hz) ending with an electric current (0.7 mA, 2 s, pulsed 25 Hz); (2) assessment of conditioned fear 24 h later in a context-dependent retention test [180 s, same box and light conditions as before (rectangular cage, 125 lx)], and (3) 2 h later in a tone-dependent retention test (180 s, 9 kHz, 75 dB SPL, pulsed 5 Hz) with different context (round cage, plane floor, 500 lx).

### Monitoring of behavior

For the Morris water maze task a computerized video system registered moving-path and duration automatically. The hardware consisted of an IBM-type AT computer combined with a video digitizer and a CCD video camera. The software used for data acquisition and analysis was EthoVision XT release 8.5 (Noldus Information Technology, Utrecht, Netherlands). In case of fear conditioning, the experiments were performed in a computerized fear-conditioning system where activity of the animal is recognized by infrared laser beams (TSE-Systems, Bad Homburg, Germany). During training and context-dependent retention test, mice were placed in a Plexiglas cage (context 1; 20 × 20 × 40 cm). Tone-dependent retention tests were performed 2 h after the contextual memory test in a novel context (context 2). Context 2 was a circular Plexiglas cage with 19.0 cm in diameter and 40.0 cm in height. Grid floor was covered by a grey plastic bottom and light level was changed as mentioned above. The fear conditioning box was thoroughly cleaned with 70 % ethanol before the placement of each mouse.

### Quantification and statistical analysis

Data were analyzed by using GraphPad Prism 6 (Graph Pad Software, La Jolla, CA, USA) or SPSS (IBM, Ehningen, Germany). Cell culture experiments were statistically analyzed by one-way ANOVA followed by Bonferroni multiple comparisons test. For the behavioral studies, data were analyzed by ANOVA for differences between the treatment conditions. Multivariate analysis of variance (two-way ANOVA) was performed for learning and memory testing. Probe trial scores within experimental groups were evaluated by a one-way ANOVA. Post hoc comparisons of the treatment were based on Student’s *t* test, difference to chance levels in quadrant occupancy by one sample *t* test. Differences were considered significant for p ≤ 0.05.

## Results

**Fig. 1:**
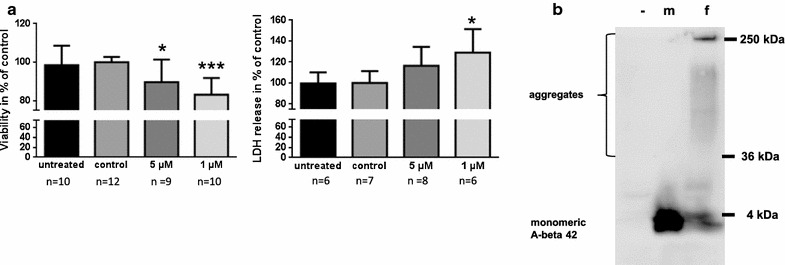
Characteristics of A-beta 42 peptides in vitro. **a** SH-SY5Y cells were treated for 24 h with A-beta 42 peptides, solvent (*control*) or pure culture medium (*untreated*). Cell viability was assessed by MTT assay (*left* graph) and by LDH release analysis (*right* graph). Values were calculated as % of control cells and are represented as mean and SD from three independent experiments (conducted in at least duplicate, n ≥ 6 as indicated). Significant differences between solvent-treated controls and A-beta 42 treated cells (one-way Anova, Bonferroni’s multiple comparisons test) are indicated by *(p < 0.05) and ***(p < 0.001). **b** A-beta peptides were incubated at 37 °C for 24 h under agitation in PBS (fibrillated, f). As controls, untreated A-beta 42 peptides (monomeric, m) and buffer with unrelated protein (trypsin, −) were used. 7.4 µg of protein each were subjected to 10 % SDS–polyacrylamide gel, transferred to nitrocellulose and detected with antibody 6E10 and anti-mouse horse radish-labeled secondary antibody

The aim of our study was to investigate if application of A-beta 42 peptides via the nasal cavity is able to evolve lesions within the mouse brain that lead to changes in learning and memory performance. Initially, we tested the peptides used in our animal study for their capability of evoking toxic effects on neuronal cells. A 24 h treatment period of human neuroblastoma cell line SH-SY5Y with 1 µM monomeric peptides resulted in about 20 % reduction of viability as compared to control cells (Fig. 1a, left graph). This was accompanied by an increase of 30 % of LDH in the cell supernatant (Fig. 1a, right graph), indicating the toxic characteristic of peptide preparation. With 5 µM we observed reduced viability of only 90 % compared to control and only in tendency enhanced secretion of LDH into the medium. This might be due to the fact that A-beta accumulates at higher dosage in the endosomal/lysosomal compartment as well as on the cell surface and is able to interfere in a toxicity-independent manner with both, the reduction reaction and integrity of the membrane (e.g. [17, 18]).

**Fig. 2:**
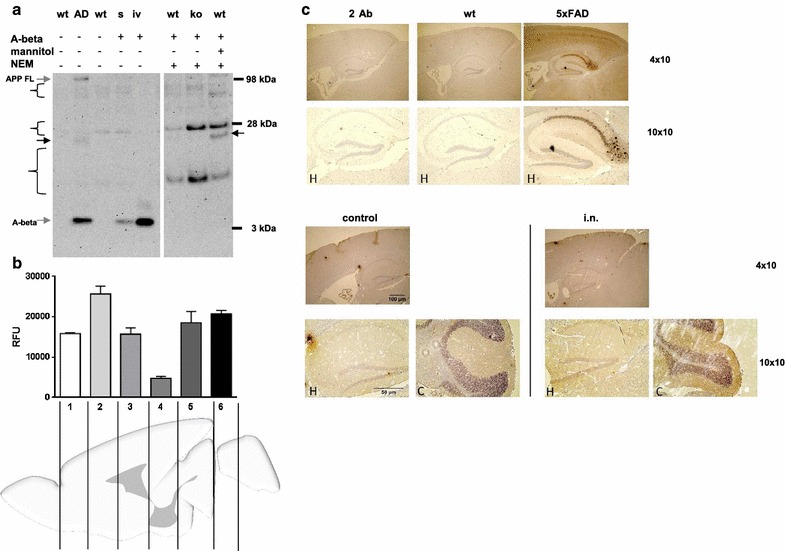
Detection of A-beta 42 peptides within brain after intranasal acute application. **a** Mice (wt: FVB/N; ko; Pgp knock-out) were treated with NEM and mannitol as indicated. Subsequently, A-beta was intranasally administered and animals were sacrificed after 1 h. Brains were dissected and proteins extracted by formic acid treatment. 20 µg of proteins were subjected to 4–12 % SDS–polyacrylamide gel and transferred to nitrocellulose membrane. Signals were obtained as described in Fig. 1. As a control, brain extracts from a 5xFAD Alzheimer model mouse (AD) and the corresponding wild type littermates (wt, C57B6/J) were used (aged 9 months). Additionally, pure A-beta (in vitro, iv) and wild type brain spiked with A-beta during the extraction process (s) were analyzed. A band potentially representing A-beta oligomers from the 5xFAD or A-beta/mannitol treated animal is depicted by an *arrow*. Unspecific signals that also occur in a “secondary antibody only” incubation (Additional file 2) are indicated by *curly brackets*). **b** For assessment of A-beta distribution upon intranasal delivery, 10 µg of TAMRA-labeled A-beta 42 peptide or water in diluent solution (control) were administered to C57Bl6/J wt mice pre-treated with mannitol (one exemplary animal for each treatment). 1 h after the application, slices from brains (cut positions indicated in the scheme) were used for detection of light emission at 580 nm in technical replicates of tissue homogenates. RFU (relative fluorescence units) were calculated by substracting values obtained from TAMRA-A-beta 42 treated animal by values measured for the control animal (mean and SD). **c** To analyze distribution of intranasally administered A-beta 42 peptides, animals were treated as described in B with unlabeled A-beta 42 peptide (i.n.) or solvent (control) and brains dissected 1 h after treatment. Staining of sagittal sections was performed with primary antibody 6E10. For comparison, a “secondary antibody only” staining (2°Ab) and samples from a wild type as well as a 5xFAD transgenic mouse (4 months of age) are shown. An overview of the midbrain region of intranasally treated mice is given (4 × 10 magnification) as well as hippocampal (H) and cerebellar (C) region in higher magnification (10 × 10)

Next, we wanted to demonstrate the ability of the peptides used to form higher molecular weight species at physiological temperature and buffer conditions. Upon 24 h incubation at 37 °C under agitation most of the monomeric peptide was included in oligomeric forms or protein species with up to about 250 kDa (exemplary western blot; Fig. 1b) which represent large aggregates [19]. Subsequently, we wanted to investigate, if intranasally administered A-beta peptides might be detectable after acute treatment in the brain. In Alzheimer model mice (Fig. 2a 5xFAD, [13, 20]), which were used for comparison, a weak band for what might represent residual full-length APP from extraction and a strong band for monomeric A-beta were detected. Both were absent in wild type mice only expressing endogenous murine APP. In wild type mice treated with A-beta via nasal route and the degradation inhibitor N-ethylmaleimide (NEM, [21]) alone, no bands corresponding to the exogenously administered A-beta peptides were observed. As it has been described that A-beta might be efficiently eliminated from brain via P-glycoprotein (P-gp [22]), the main export transporter of the blood brain barrier, we tested P-gp knock-out mice for a potential detectable accumulation of the exogenous peptides in brain tissue. Similarly to the NEM treated wild type, no additional bands occurred in western blotting experiments. Only samples from mice pre-treated with mannitol, which relaxes blood–brain barrier after* i.p.* injection [14], showed bands detectable with antibody 6E10 that might reflect A-beta aggregation products after tissue extraction: e.g. a band of about 25 kDa that is also present in 5xFAD mouse brain tissue preparation but not in untreated wild type mice was visible (Fig. 2a, arrows). In regard to the fact, that tissue spiked with monomeric A-beta showed only about 30 % of the expected signal derived from A-beta incubated without tissue, retrieval rate is rather low and might severely impair A-beta detection without further labeling of the peptide (Fig. 2a, lane s compared to lane iv). In sum, we decided to pre-treat mice with mannitol before nasal delivery of A-beta 42 peptides in behavioral testing. To demonstrate the potential of A-beta peptides to enter also deeper regions of the brain after intranasal application, we treated an exemplary wild type mouse with TAMRA-labeled A-beta 42 and a control mouse with solvent and investigated six different slices obtained from the brains according to fluorescence signal: the highest fluorescence intensity was obtained from a slice containing cortical regions (2, Fig. 2b). The lowest signal was measured from samples derived from Tectum/Tegmentum region but fluorescence was detectable throughout the brain also reaching cerebellar tissue (6, Fig. 2b). This indicates that the fluorescence-labeled peptide enters the brain and is present in brain regions needed for learning and memory function already shortly after application. We therefore assume that also the unlabeled peptides would be delivered in analogy and therefore have an impact on brain function. To make sure that not only the fluorophore is delivered throughout the brain but also the coupled peptide, we performed ELISA targeting the exogenously administered human A-beta 42 and obtained values ranging from about 700 pg/ml in the first two slices down to 15 pg/ml after correction for untreated control background (Additional file 1).

Additionally, we stained sagittal brain sections from wild type C57B6/J mice sacrificed 1 h after transnasal delivery of A-beta 42 peptides with antibody 6E10 (Fig. 2c). A 4 month old 5xFAD mouse served as a positive control and showed cortical as well as even more pronounced staining of the hippocampus, while analysis of secondary antibody control and the respective wild type littermate did not result in specific staining signals. In the peptide-treated animal (i.n., Fig. 2c), no distinct deposits as observed in the genetic AD model occurred. However, brain slices seemed to display a slight increase in overall diffuse staining as shown for hippocampal as well as cerebellar region, indicating presence of antigen.

**Fig. 3:**
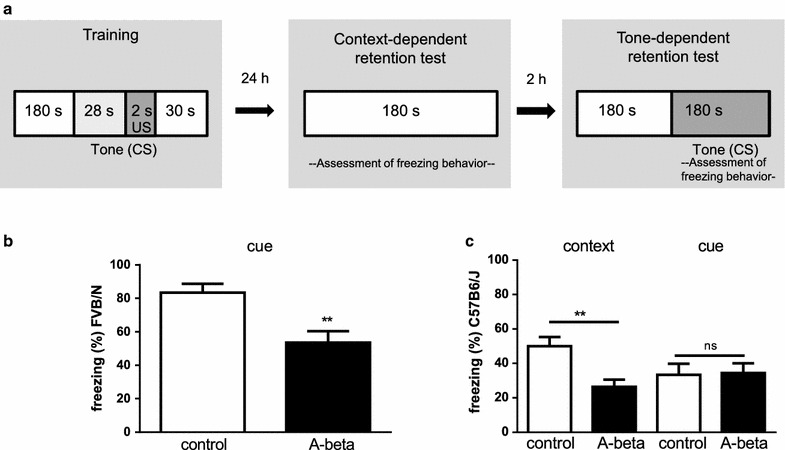
Impact of transnasally delivered A-beta peptides on fear conditioning memory. **a** Schematic representation of the fear conditioning experiments with stimulus sequences, and variable training-test intervals. Animals were treated for three consecutive days with A-beta 42 peptides administered to the nose or with solvent control. On the second application day, mice underwent one training session with 180 s habituation, presentation of the conditioned stimulus (CS, tone, 30 s) with a mild foot shock within the last two seconds (US: unconditioned stimulus). The following day (third treatment day), mice were subjected to (1) context-dependent retention test (similar test situation without any stimulus), and (2) tone-dependent retention test (new context with CS presentation). **b** Cue-dependent fear conditioning related analysis in FVB/N mice either with A-beta 42 (*black bar*) or solvent treatment (*white bar*). The diagram represents total time of freezing behavior in the conditioned stimulus presentation (in %) ± SEM (n = 5 for each group) (**p < 0.01). **c** Fear conditioning related analysis in C57Bl6/J mice treated with A-beta 42 peptides (*black bars*, n = 7) or control animals (*white bars*, n = 13). The diagram represents total time of freezing behavior (%) ± SEM. Significant effects (p < 0.01) are indicated by **

For in vivo effects of intranasal A-beta 42 we investigated its potential influence on learning and memory in two different laboratory mouse strains. At first we studied behavior in a fear conditioning task with respect to context- and cue-retention. FVB/N mice treated with A-beta 42 were significantly impaired compared to solvent treated controls in the cue-retention condition (Fig. 3b). Freezing-reaction after tone presentation was about 1.5 times lower in A-beta treated mice indicating a significant deficit due to the treatment. Control mice did not show appropriate freezing behavior in context-retention under the chosen experimental parameters. This is consistent with former reports [23, 24] and might rely on presence of mutations in the Pde6b or deletion in the CP49 gene (implicated in retinal degeneration or establishing of lens fiber network) in this strain [25, 26]. Therefore, changes due to peptide-treatment in context-conditioning were not measurable in FVB/N mice. To make sure that impaired behavior based on A-beta treatment is not restricted to a single genetic background, we also included another commonly used mouse strain which is most often the background of transgenic Alzheimer model mice: C57Bl6/J mice treated with A-beta 42 showed a strong impairment in the context-retention condition by a reduction of freezing time compared to controls of about 50 % (Fig. 3c). However, in the cue-condition no significant difference was seen (control: 33.37±6.51 %; A-beta 42: 34.37±5.71 %; p = 0.921) but freezing of control mice also appeared weaker due to tone presentation than due to context (p = 0.059). Such peculiarities regarding behavioral tasks have already been reported in literature for different mouse strains [27].

**Fig. 4:**
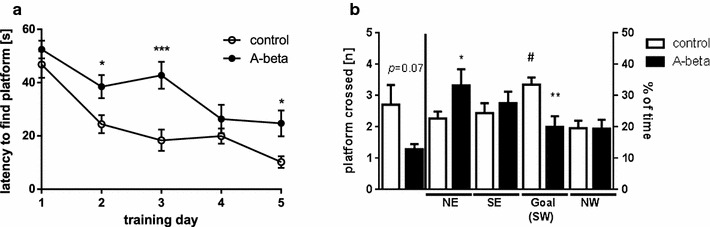
Influence of intransally administered A-beta peptides on Morris water maze task in C57B6/J mice. **a** Learning during training days one to five is displayed by the time to find the submerged platform for mice treated with A-beta 42 peptides (*closed circles*, n = 10) or with solvent (*open circles*, n = 14) Significant daily differences (Student’s *t* test) are indicated by *(p < 0.05) and ***(p < 0.001). **b** Memory related behavior based on crossing the former platform location or time spent in the quadrant searching the platform at the probe trial (day 6). Crossings are given as total numbers (Student’s *t* test; *p < 0.05, *left* part of the graph) and time spent in the respective quadrant (*NE* North East, *SE* South East, *SW* South West, *NW* North West) is given in % of total time (60 s). *White bars* represent group mean ± SEM of control-treated animals whereas *black bars* represent values obtained for A-beta 42 peptide treated animals (*p < 0.05; **p < 0.01, one-way ANOVA, Sidak’s multiple comparisons test; #p < 0.05 indicates dwell times above chance level (25 %) one sample *t* test)

Another behavioral domain characteristically impaired in Alzheimer model mice is the spatial memory investigated by the Morris water maze task (MWM, [28]). Due to the fact that FVB/N mice were known to be unsuitable in this task [23] we restricted the investigation to the C57Bl6/J strain. With respect to activity of the mice while performing the MWM task no differences between treatment conditions were seen (swim speed: 13.0 cm/s in control mice; 12.4 cm/s in A-beta treated mice; p = 0.72) allowing for evaluation of cognitive performance. With respect to spatial orientation, untreated mice were able to learn the position of the submerged platform within 5 days indicated by a significant reduction in time to reach the platform. A-beta 42 treated mice were also able in general to improve over the 5 days training period (Fig. 4a) but exhibited deficits in finding the platform indicated by a significant impairment on days two, three and five. The significance of the interaction term of a two-way ANOVA (AxB F_(4,88)_ = 2.59; p = 0.05) displayed the impact of A-beta treatment as the learning developed differentially as compared to control mice [two-way ANOVA, factor A time, F_(4,88)_ = 26.49; p < 0.0001, factor B treatment F_(1,22)_ = 10.84; p = 0.01].

While in control mice time to reach the platform decreased from 46.83 ± 4.95 to 10.16 ± 2.17 s within the 5 days; the mice receiving intranasal A-beta 42 treatment needed at each of the four time points at least 24.71 ± 4.88 s to find the submerged platform. Similar results have been reported age-dependently for mice that harbor diffuse beta-amyloid deposits but do not form plaques [29]. For investigation of memory performance, the platform was removed and similar as during learning behavior, A-beta 42-treated mice needed significantly longer swimming times to find the former platform location (data for day 6 not shown). The number of crossings of the supposed and former platform location was also reduced in A-beta-treated animals but did not reach statistical significance (Fig. 4b). However, when analyzing time spent in the respective quadrants, which is indicative of memorizing its location dwell times above chance level in the goal quadrant were only seen in solvent-treated mice (Fig. 4b). This indicates that A-beta-treated mice were not able to recall the position of the platform properly. Both behavioral aspects—fear conditioning as well as Morris-water maze learning—have been demonstrated earlier to be A-beta sensitive [30–32]. When brain hemispheres of mice from behavioral tasks were subsequently used for A-beta 42 ELISA, no detectable amount above background signal was measured in samples derived from peptide treated mice (Additional file 3). We therefore have to assume that although peptides were probably already cleared from the brain after the behavioral tests, they were able to induce cellular reactions during the 3 days of treatment that in consequence led to behavioral impairment. Similar findings have been reported for alteration of ERK signaling upon local brain infusion with A-beta 42 peptides [33].

## Discussion

Confirmed post mortem AD pathology shows accumulation of extracellular A-beta in plaques and intracellular neurofibrillary tau tangles in the brain. The involvement of A-beta in AD is a prerequisite to the significance of peptide production or clearance to AD. It should be noted that A-beta is a naturally occurring endogenous peptide that may have normal physiological functions. For example, it has been shown that picomolar concentrations of A-beta increased LTP resulting in improved synaptic plasticity and memory [34–36]. Therefore, pathology associated with A-beta has to be related to its aberrant accumulation/aggregation. Familial forms of early-onset AD are caused by mutations in APP, PS1, or PS2 (for a personal view on the discovery of those genes see [37]) or through increased copy number of APP [38–40]. This enabled pre-clinical research to establish multiple animal models that display various functional deficits either related to tau or A-beta pathology at various stages and age [2]. While the familial genetics clearly point to A-beta as a critical factor in the etiology of AD, it is conceivable that the much more common sporadic form of the disease (i.e., LOAD) has a distinct origin potentially independent from A-beta production. Nevertheless, several lines of evidence suggest otherwise [41, 42]. The differences between FAD and LOAD made it difficult for pre-clinical research in development of animal models to investigate mechanisms underlying LOAD. Some animal species such as aging *Octodon degu* or primates spontaneously develop AD pathology indicated by plaques or tauopathy (reviewed in [43]) but research in those models is restricted due to economic as well as ethical reasons. Exogenous administration of A-beta in rodent brain resulted in different outcomes, depending for example on site of application [44, 45]. Various approaches have been made with A-beta elevating drugs or modulators of tauopathies to establish spontaneous AD models: for example slow injection of thiorphan, a neprilysin inhibitor, by a osmotic minipump into the hippocampus led to decrease in learning and memory performance in rats after 4 weeks [46]. Administration of Wortmannin, an activator of GSK3beta, in the lateral ventricle of rats resulted in hyperphosphorylation of tau [47]. As thiorphan is able to inhibit not only A-beta degradation but also that of endogenous enkephalins [48] and Wortmannin acts via PI3 kinase/PKB/Akt pathway (short overview in [49]), side effects apart from direct interference with Alzheimer-related targets have to be considered in such efforts.

Our approach presented here provides good evidence for the investigation of A-beta 42 mediated deficits not caused by genetic pre-disposition, after surgical manipulation or by treatment with pharmacological compounds with potential side effects. Intranasal application has been shown several times to be a painless, harmless way to deliver biologics or treat disease related symptoms (overview in: [50]). An intranasally administered antibody against A-beta peptides was found to penetrate the brain within 2 h and to spread throughout the brain within 12 h post injection [51]. Detection of the HRP-labeled antibody within the brain parenchyma was observed as early as 6 h post injection in the vicinity of the 3rd ventricle, midbrain and hippocampus in these experiments. However, the peptide we used here is obviously smaller and we pre-treated animals with mannitol. Delivery of drugs via the nose has been reported to generally circumvent the blood brain barrier which is transiently disrupted by treatment with hyperosmolaric sugar solutions such as mannitol. Nevertheless, effects of mannitol on nasal and airway epithelium has been described that might further support entry of the peptide into the brain [52–54]. As the animals were not perfused in our study prior to tissue harvesting, we cannot rule out in principle that observed signals for native or fluorophore-labelled A-beta peptides might derive from blood comprised in tissue homogenates. Intravenously injected Cy5.5 labelled A-beta 40 for instance colocalized with UEA-1 stained cerebral vessels 8 h post injection [55].

However, functional deficits in two learning- and memory-related behavioral tasks pointed towards the efficacy of intranasal treatment with A-beta 42. Likewise genetic FAD mouse models [56] mice in our study displayed deficits in MWM as well as in fear conditioning. A-beta 42 had a significant impact on hippocampus-dependent spatial learning and memory and context-dependent fear conditioning (Figs. 3c, 4; [57, 58]) and additional amygdala-related memory function (Fig. 3b; [58, 59]) as has been shown in FAD mouse models [56]. Those deficits were already visible after 3 days of treatment with the peptide while in AD transgenic model mice behavioral deficits occur mostly after plaque deposition at an earliest time point of 1–2 months of age [2]. In an AD mouse model with genetic cause of origin, it has been reported that plaques are able to grow within 24 h and elicit local microglia activation within 1–2 days [60]. In our study, we administered 10 µg A-beta 42 daily which theoretically would result in a concentration of about 30 ng peptide per mg tissue and thus is three times higher than levels found in the double transgenic APP/PS1 mouse model (e.g. [61]). Taking into account that A-beta is quickly removed from brain via transporters of the blood brain barrier or degradation (half life time of 2 h reported in [62]) and probably not all peptide is resorbed via the nasal epithelium, we would assume that effective A-beta levels in the brain were in fact lower and observed effects are based mainly on soluble A-beta species. In this regard it would be of interest, if A-beta 40 and 42 would differ in outcome intensities of behavioral effects because clearance of the 40 amino acid peptide from brain tissue has been described to be faster after ivc [63] and also architecture of fibrils and plaques differ in between both peptide species [64].

In sum, our model allows investigation of acute A-beta dependent influences within the brain. Nevertheless, it has its limitations due to the fact that sporadic AD develops over decades within human life [65]. This has consequences regarding e.g. long-term inflammatory events, occurrence of compensatory mechanisms or even brain atrophy at later stages that might obviously not be observed in such a model of direct application. Additionally, our treatment might not reflect routes of spreading characteristic for spontaneous AD. Intranasal administration utilizes three main delivering pathways for material to the brain: the olfactory [66], the rostral migratory stream [67], and trigeminal routes (e.g. [68]). This in consequence leads to a fast delivery that nearly targets all brain areas from olfactory bulbs throughout the hindbrain. In AD, the commonly-received concept assumes an origin within the transentorhinal and entorhinal cortex from where pathology spreads slowly throughout the entire brain. Additionally, within the last years, propagation of AD pathology via a neuronal cell-to-cell transfer has been suggested by cell culture and animal experiments [69, 70]. In transnasal delivery, however, trans-neuronal and para-neuronal pathways seem to contribute depending on the administered drug formulation [71]. As we did not investigate the spreading of the exogenously applied peptides within the brain further, we cannot speculate yet on the route taken.

## Conclusions

In sum, our results seem promising regarding a model for acute alterations caused by A-beta peptides in the mouse that might have the potential to mimic aspects of LOAD. Nevertheless, there remain some questions in regard to optimal dosage, treatment duration, age of treatment onset, efficacy of epithelial transport and multiple other effects of intranasal delivery of A-beta 42, which have to be answered in future investigations.

## Additional files

10.1186/s12868-016-0280-9 Analysis of A-beta 42 peptides from acute transnasally treated mice. An exemplary wild type animal was treated with TAMRA-labeled A-beta 42 peptides and a control mouse with solvent administered to the nose (see Figure 2B). Brains were dissected 1 h later and homogenized as described. Extraction supernatant was subjected to human A-beta 42 ELISA (IBL) as recommended by the vendor. Values obtained for A-beta 42 containing slices were corrected for background values obtained from slices of solvent-control. Note that signal intensities for the six slices deviate from the fluorescence-based A-beta 42 measurement. This might indicate a partial liberation of the fluorophore from the peptide or be due to interference of tissue contents with the two different measurement techniques. Nevertheless, human A-beta 42 peptides were detectable throughout all of the six slices upon intranasal application also by peptide-directed ELISA.
10.1186/s12868-016-0280-9 Specificity control for antibody 6E10. 20 μg of proteins from brain tissue of wt or AD model mouse (5xFAD) were subjected to 4-12 % SDSpolyacrylamide gel and transferred to nitrocellulose membrane. Signals were obtained by combining antibody 6E10 and secondary HRP-coupled antibody (left) or by secondary antibody only (right).
10.1186/s12868-016-0280-9 Analysis of A-beta 42 peptides from transnasally treated mice. Animals were treated for three consecutive days with A-beta 42 peptides administered to the nose or with solvent control. Brains were dissected after behavioral task performance of the mice, stored at -80 °C and extracted with 70 % formic acid. 100 μl extraction supernatant from solvent (n = 3) or peptide-treated (n = 4) mice were subjected to human A-beta 42 ELISA (IBL) as recommended by the vendor. All measured values were at or below the lowest standard (red dots). As a positive control we used brain extract from an age-matched 5xFAD mouse (green triangle).

## Abbreviations

A-beta: amyloid beta peptide
AD: Alzheimer’s disease
APP: amyloid precursor protein
BBB: blood brain barrier
FAD: familial Alzheimer’s disease
Ko: knock-out
LOAD: late onset Alzheimer’s disease
MWM: morris water maze
Wt: wild-type
