# Supplementary material for: Transnasal delivery of human A-beta peptides elicits impaired learning and memory performance in wild type mice
Source: BMC Neurosci. 2016 Jul 4;17:44. doi: 10.1186/s12868-016-0280-9 (PMC4932715; doi:10.1186/s12868-016-0280-9)
Supplement: Supplementary file 1 — 10.1186/s12868-016-0280-9 Analysis of A-beta 42 peptides from acute transnasally treated mice. An exemplary wild type animal was treated with TAMRA-labeled A-beta 42 peptides and a control mouse with solvent administered to the nose (see Figure 2B). Brains were dissected 1 h later and homogenized as described. Extraction supernatant was subjected to human A-beta 42 ELISA (IBL) as recommended by the vendor. Values obtained for A-beta 42 containing slices were corrected for background values obtained from slices of solvent-control. Note that signal intensities for the six slices deviate from the fluorescence-based A-beta 42 measurement. This might indicate a partial liberation of the fluorophore from the peptide or be due to interference of tissue contents with the two different measurement techniques. Nevertheless, human A-beta 42 peptides were detectable throughout all of the six slices upon intranasal application also by peptide-directed ELISA. [file 12868_2016_280_MOESM1_ESM.pdf]

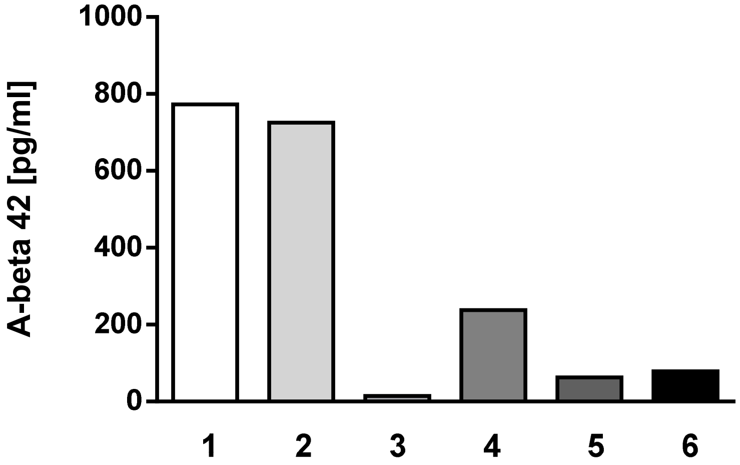

Analysis of A-beta 42 peptides from acute transnasally treated mice.

An exemplary wild type animal was treated with TAMRA-labeled A-beta 42 peptides and a control mouse with solvent administered to the nose (see Figure 2B). Brains were dissected 1h later and homogenized as described. Extraction supernatant was subjected to human A-beta 42 ELISA (IBL) as recommended by the vendor. Values obtained for A-beta 42 containing slices were corrected for background values obtained from slices of solvent-control. Note that signal intensities for the six slices deviate from the fluorescence-based A-beta 42 measurement. This might indicate a partial liberation of the fluorophore from the peptide or be due to interference of tissue contents with the two different measurement techniques. Nevertheless, human A-beta 42 peptides were detectable throughout all of the six slices upon intranasal application also by peptide-directed ELISA.
