# Supplementary material for: Transnasal delivery of human A-beta peptides elicits impaired learning and memory performance in wild type mice
Source: BMC Neurosci. 2016 Jul 4;17:44. doi: 10.1186/s12868-016-0280-9 (PMC4932715; doi:10.1186/s12868-016-0280-9)
Supplement: Supplementary file 2 — 10.1186/s12868-016-0280-9 Specificity control for antibody 6E10. 20 μg of proteins from brain tissue of wt or AD model mouse (5xFAD) were subjected to 4-12 % SDSpolyacrylamide gel and transferred to nitrocellulose membrane. Signals were obtained by combining antibody 6E10 and secondary HRP-coupled antibody (left) or by secondary antibody only (right). [file 12868_2016_280_MOESM2_ESM.pdf]

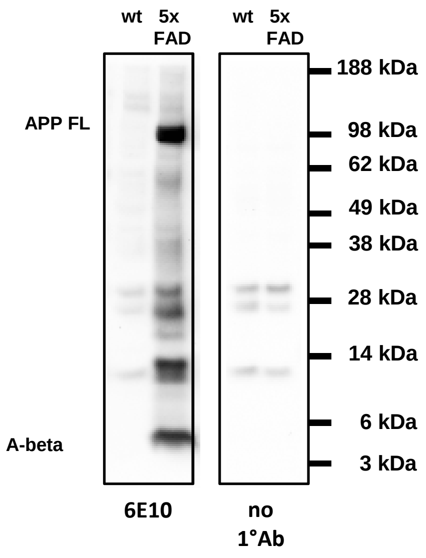

Specificity control for antibody 6E10.
20 µg of proteins from brain tissue of wt or AD model mouse (5xFAD) were subjected to 4-12% SDS-polyacrylamide gel and transferred to nitrocellulose membrane. Signals were obtained by combining antibody 6E10 and secondary HRP-coupled antibody (left) or by secondary antibody only (right).
