# Supplementary material for: Transnasal delivery of human A-beta peptides elicits impaired learning and memory performance in wild type mice
Source: BMC Neurosci. 2016 Jul 4;17:44. doi: 10.1186/s12868-016-0280-9 (PMC4932715; doi:10.1186/s12868-016-0280-9)
Supplement: Supplementary file 3 — 10.1186/s12868-016-0280-9 Analysis of A-beta 42 peptides from transnasally treated mice. Animals were treated for three consecutive days with A-beta 42 peptides administered to the nose or with solvent control. Brains were dissected after behavioral task performance of the mice, stored at -80 °C and extracted with 70 % formic acid. 100 μl extraction supernatant from solvent (n = 3) or peptide-treated (n = 4) mice were subjected to human A-beta 42 ELISA (IBL) as recommended by the vendor. All measured values were at or below the lowest standard (red dots). As a positive control we used brain extract from an age-matched 5xFAD mouse (green triangle). [file 12868_2016_280_MOESM3_ESM.pdf]

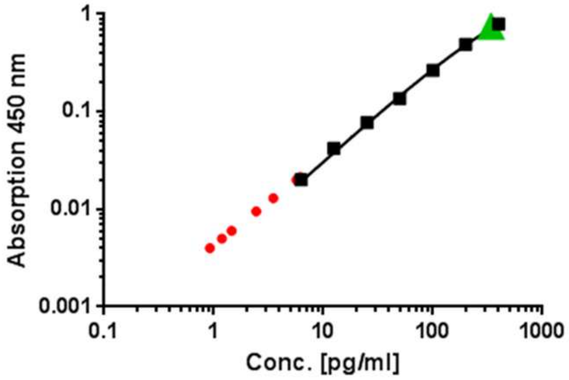

Analysis of A-beta 42 peptides from transnasally treated mice.
Animals were treated for three consecutive days with A-beta 42 peptides administered to the nose or with solvent control. Brains were dissected after behavioral task performance of the mice, stored at -80 C and extracted with 70% formic acid. 100 µl extraction supernatant from solvent (n=3) or peptide-treated (n=4) mice were subjected to human A-beta 42 ELISA (IBL) as recommended by the vendor. All measured values were at or below the lowest standard (red dots). As a positive control we used brain extract from an age-matched 5xFAD mouse (green triangle).
